# The risk of obesity by assessing infant growth against the UK-WHO charts compared to the UK90 reference: findings from the Born in Bradford birth cohort study

**DOI:** 10.1186/1471-2431-12-104

**Published:** 2012-07-23

**Authors:** William Johnson, John Wright, Noël Cameron

**Affiliations:** 1 Division of Epidemiology & Community Health, School of Public Health, University of Minnesota, 1300 2nd Street S, Suite #300, Minneapolis, MN, 55455, USA; 2 Bradford Institute for Health Research, Bradford Royal Infirmary, Duckworth Lane, BD9 6RJ, UK; 3 Centre for Global Health and Human Development, Loughborough University, Leicestershire, LE11 3TU, UK

**Keywords:** Growth charts, Postnatal growth, Infant, Obesity, Longitudinal studies

## Abstract

**Background:**

The new growth charts in the UK, the UK-WHO charts, comprise prescriptive data from the WHO standard between two weeks and four years of age. Little is known about the development of obesity risk in normal UK infants, who are necessarily not fed according to the WHO recommendations and do not live in constraint-free environments (the selection criteria of the WHO standard source sample), using the new charts. Here, we investigated infant growth trajectories and traits indicative of childhood obesity using the UK-WHO charts, with the aim to clearly document the implications of adopting the new charts on UK growth monitoring practice.

**Methods:**

Mixed effects models were applied to serial weight and length data from 2181 infants (1187 White; 994 Pakistani) in the Born in Bradford birth cohort study to produce curves from 10 days to 15 months of age. Individual monthly estimates were converted to Z-scores and were plotted by sex and ethnic group. The relative risks (RR) of traits indicative of childhood obesity, including high BMI and rapid weight gain, using the UK-WHO charts compared to the previously used UK90 reference were calculated for all infants together and for White and Pakistani infants separately.

**Results:**

Both ethnic groups demonstrated patterns of growth similar to the UK-WHO charts in length but not in weight. The resulting pattern for BMI was remarkable, with an average gain of 1.0 Z-score between two and 12 months of age. The UK-WHO charts were significantly (p < 0.05) more likely than the UK90 reference to classify BMI above the 91^st^ centile after age six months (RR 1.427-2.151) and weight and BMI gain between birth (one month for BMI) and 12 months of age greater than two centile bands (RR 1.214 and 1.470, respectively).

**Conclusions:**

The change to the UK-WHO charts means that normal UK infants risk being diagnosed as being on a trajectory toward childhood obesity. National estimates of obesity will have to be recalculated for previous years to allow longitudinal comparison. The new charts do not allow a focused prevention effort for targeting programmes at infants most at risk of becoming obese, because the use of the 91^st^ or 98^th^ centile on the UK-WHO charts will identify many more infants as being at risk than the same centiles on the UK90 reference. Now more than ever, research is needed to develop a large scale childhood obesity prevention programme which could ideally be integrated with routine infant growth monitoring practice.

## Background

Routine growth assessment is a fundamental part of the monitoring of child health in the United Kingdom (UK) [1]. Growth in weight, length, and head circumference is measured by child health practitioners during infancy at 10 to 14 days and six to eight weeks of age; weight is measured again at 12 months and 24 to 30 months of age [2]. The primary reason for these assessments has traditionally been for the identification of growth faltering. However, recent increases in the prevalence of childhood obesity [3,4], and the identification of rapid infant weight gain as a determinant of later obesity [5-7], have emphasised the need for routine growth assessment to identify infants on a trajectory toward obesity.

The normality of the pattern of growth of an infant is determined by comparison to a growth chart. In the UK, the current growth charts are a combination of the UK90 *reference*[8] and the World Health Organisation (WHO) 2006 child growth *standard*[9]. The former is a true growth reference depicting the normal growth of children, whilst the WHO standard depicts the *optimal* growth of children who were specifically selected for inclusion in the chart source sample because they were exclusively or predominantly breastfed until at least four months of age in environments free from any socio-economic constraint to their growth [10]. Goldstein and Tanner [11] provide good discussion on the difference between a growth standard and a growth reference. Adopted for practice in 2009, the new charts in the UK are called the UK-WHO child growth charts (http://www.rcpch.ac.uk/growthcharts); they combine recalculated UK90 reference birth data [12] with the WHO standard data from ages two weeks to four years.

The use of the WHO standard data for growth assessment of UK infants presents a problem of interpretation. That dilemma is epitomised by the fact that the majority of UK infants are not fed according to the WHO recommendations and do not live in constraint-free environments [13-16]. The similarity of their growth to that depicted in the WHO standard is thus not guaranteed. Indeed patterns of growth of infants from two UK studies used to test the WHO standard for adoption in the UK identified the fact that whilst growth in length was similar to the WHO standard, growth in weight differed significantly, demonstrating initial faltering followed by acceleration through the centiles [17]. It was explained that this acceleration (in weight but not length) would “support efforts to avoid future childhood obesity” because more infants would be identified as having a high body mass index (BMI) [17].

This study builds on knowledge from the one published study [17] that presented data at cross-sectional time points by 1) assessing the conformity of the weight and length longitudinal infant growth curves of two ethnic groups in the UK to the UK-WHO charts, and 2) determining the risk of infant growth traits indicative of later obesity using the UK-WHO charts compared to the previously used UK90 reference charts. The paper provides novel information about the development of obesity risk in UK infants using the UK-WHO charts.

## Methods

### Sample

The sample comprised 2181 singleton term infants participating in the Born in Bradford birth cohort study of whom 1187 (564 girls) were of White British ancestry and 994 (474 girls) were of Pakistani ancestry. Born in Bradford has been described in detail elsewhere [18], but in summary is a study tracking the growth and health of a cohort of 13,000 individuals born between 2007 and 2011 in the post-industrial city of Bradford, UK.

The sample was selected on the basis of having good serial weight and length data for growth curve modelling (see below). Scatter plots of anthropometry against age for the sample and for all White and Pakistani Born in Bradford term infants with anthropometric data (n = 6839 for weight, n = 6603 for length) showed that the distributions were similar. In addition, there was no significant difference in the sex and ethnic composition of the sample compared to all those infants with (weight and length) data using Chi-squared tests (p-values > 0.6).

Ethical approval was granted by Bradford Research Ethics Committee on the 16th May 2007, and research governance approval was provided by Bradford Teaching Hospitals Trust on the 26th March 2007.

### Data

Measured birth weight was obtained from routine hospital records. Child health practitioners measured the weight and length of the infants in the community, as part of routine practice, using standard measurement procedures [19] following a programme of training by an acknowledged expert (NC). During the period when these data were collected (2007 and 2008) the target assessment ages were 10 to 14 days, six to eight weeks, seven to nine months, and 18 to 24 months of age. In reality, however, measurements occurred at non-standard ages [20]. In total, 14,283 serial weight measurements and 8,052 serial length measurements were collected on the 2181 infants. The reliability of these routine measurements was assessed through a quality control study, which reported inter-observer mean technical errors of measurement of 21 g for weight and of 0·6 cm for length [21].

### Growth curve modelling

To best utilise the longitudinal data, mixed effects growth models were applied to weight and length data collected from 10 days to 15 months of age to produce growth curves for each sex and each ethnic group. These models are an advancement over conventional linear regression approaches because they effectively handle the hierarchical nature of serial growth data to essentially predict individual curves whilst simultaneously estimating an average or mean curve that has been adjusted for between individual variation [22]. Outside the selected age range, the data were too infrequent to model. Infants had a minimum of three serial measurements for each dimension, with at least one occurring before age three months and at least one occurring after age six months. In total, 12,784 weight measurements, with an average of 5.9 per infant (range 3 to 44) over an average of 0.7 years (range 0.5 to 1.2), and 7,151 length measurements, with an average of 3.3 per infant (range 3 to 13) over an average of 0.7 years (range 0.4 to 1.1), were modelled.

The Berkey-Reed [23] 1^st^ order function provided a better fit for both the weight and length data than other tested structural and non-structural models. When expressed as a mixed effects model, the Berkey-Reed function can be given as:

(1) $${\text{y}}_{\text{ij}}=\beta_{0\text{j}}+\beta_{1\text{j}}\left({\text{x}}_{\text{ij}}\right)+\beta_{2\text{j}}\text{ln}\left({\text{x}}_{\text{ij}}\right)+\beta_{3}1/\left({\text{x}}_{\text{ij}}\right)$$

(2) $$\beta_{0\text{j}}=\beta_{0}+{\text{u}}_{0\text{j}}$$

(3) $$\beta_{1\text{j}}=\beta_{1}+{\text{u}}_{1\text{j}}$$

(4) $$\beta_{2\text{j}}=\beta_{2}+{\text{u}}_{2\text{j}}$$

Where, the outcome y is the size of infant j at occasion i, x is age, β_0_ is the intercept, and β_1_-β_3_ are regression coefficients that describe the shape of the curve. β_0_-β_2_ have mixed effects that comprise a sample average fixed effect (i.e., β) and a subject specific random effect (i.e., u); the fixed effects together describe the mean curve and the random effects are individual departures from the intercept and slope of that curve. Mean residuals for each month of age were within ±16 g for weight and ±0·08 cm for length. Ethnic group and sex were fitted only as main effects (i.e., an up or down shift in the entire curve for one group relative to the other group) because tested interactions with the three slope parameters of the Berkey-Reed function were not significant (p-values > 0.1). Further, we fitted separate models for each sex and ethnic group to confirm the shape of our curves to those produced when only considering data from one sex and ethnic specific group. Models were fitted using xtmixed in Stata (College Station, Texas, United States of America) allowing the intercept and first two slope parameters of the Berkey-Reed function to have random effects (as shown in the above equation).

Using the estimates of the fixed and random effects, individual estimates of weight and length at each month of age between one and 12 months were calculated. Using these values, monthly BMI values were calculated for each infant.

### Z-scores

Observed birth weight, and the monthly estimated weight, length, and BMI values between one and 12 months of age were converted to Z-scores according to the UK-WHO charts data using the excel add-in LMSgrowth (http://www.healthforallchildren.co.uk). To provide a cross-sectional data point at the end of infancy, observed weight and length, and thus BMI, data at 24 months of age (± one month) were available on 751 infants (332 White (168 girls); 419 Pakistani (214 girls)); these data were also converted to Z-scores according to the UK-WHO charts. 421 of these infants had not met the criteria for growth modelling and so were not in the sample of 2181 infants. Plots of mean weight, length, and BMI Z-scores by sex and ethnic group were produced; these plots are shown here instead of those of the growth curves against the UK-WHO charts because they allow easier comparison between dimensions and sexes.

Z-scores based on the UK90 reference data were calculated for subsequent analyses.

### Obesity risk

Our second aim was to determine the risk of infant growth traits indicative of later obesity. The traits described here may be used to define excess relative weight or weight growth in infancy but they are also known risk factors for childhood obesity [5-7]. The percentages of infants classified as overweight using each chart (i.e., BMI > +1.34 Z-scores = 91^st^ centile) was calculated at each month of age between one and 12 months, and also at age 24 months. The same was done for obesity (i.e., BMI > +2.05 Z-scores = 98^th^ centile). Rapid infant weight gain and extremely rapid infant weight gain were defined as a difference between Z-scores at 12 months and Z-scores at birth > +0.67 Z-scores and > +1·34 Z-scores, which is equivalent to upward crossing through one or two centile bands, respectively. The same approach was used to calculate rapid and extremely rapid BMI gain between one and 12 months of age. This methodology does not account for regression to the mean [24], but does reflect the actual pattern of change that child health practitioners will observe. Relative risks of these traits using the UK-WHO charts compared to the previously used UK90 reference charts were calculated and are shown for all infants together, because there were no sex differences, and for White and Pakistani infants separately, because there were some noticeable ethnic group differences.

## Results

The mixed effects growth models showed that girls were consistently 0.25 kg lighter and 1.35 cm shorter than boys over the age range being studied, and Pakistani infants were consistently 0.24 kg lighter and 0.39 cm shorter than White infants (p-values < 0.001) (data not shown).

### Growth according to the UK-WHO charts

**Figure 1:**
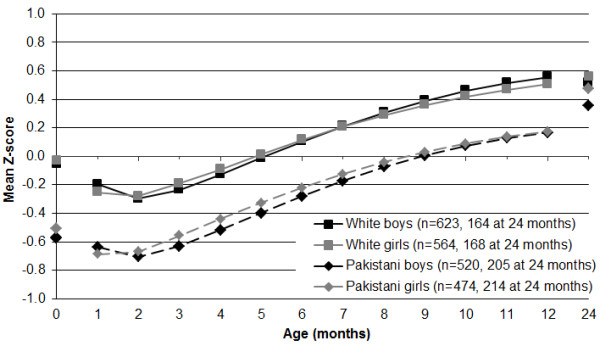
Mean weight-for-age Z-scores for 2181 Born in Bradford infants according to the UK-WHO child growth charts.

The patterns of change in weight from birth to age 12 months were similar for White and Pakistani infants (Figure 1). Both ethnic groups and both sexes demonstrated an initial period of growth faltering for the first two months and then a period of apparent accelerated growth. Mean values for Pakistani infants were consistently about 0.4 Z-scores below those of White infants. White infants reached a nadir of −0·3 Z-scores at two months of age and a maximum of +0·5 Z-scores at 12 months, compared to −0·7 and +0·2 Z-scores for Pakistani infants.

**Figure 2:**
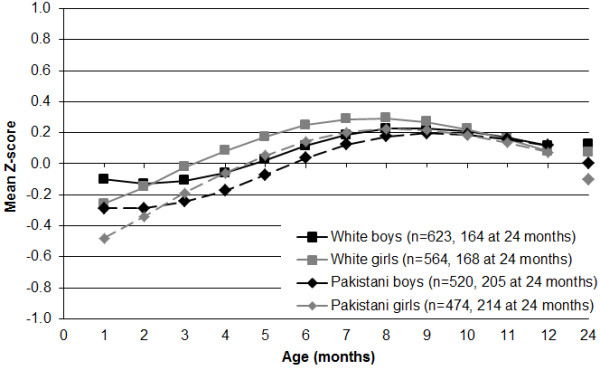
Mean length-for-age Z-scores for 2181 Born in Bradford infants according to the UK-WHO child growth charts.

Growth in length deviated from the UK-WHO charts to a lesser extent, generally varying between ±0·3 Z-scores, and was more similar for both ethnic groups, with lows for White and Pakistani infants of −0·2 and −0·4 Z-scores at age one month respectively, highs of +0·2 Z-scores at age eight months, and values within ± 0·1 Z-score of the 50^th^ centile at 12 and 24 months of age (Figure 2).

**Figure 3:**
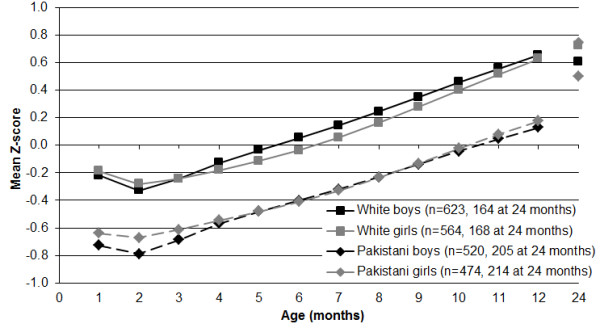
Mean BMI-for-age Z-scores for 2181 Born in Bradford infants according to the UK-WHO child growth charts.

The resulting pattern of change in BMI was quite remarkable in that, with the expected consistent difference of about 0·4 Z-scores between ethnic groups and no real differences between sexes, BMI hit a low point at age two months of −0·3 Z-scores for White infants and −0·7 Z-scores for Pakistani infants, but then increased by almost 1·0 Z-score during the next 10 months in both ethnic groups to reach > +0·6 Z-scores for White infants and almost +0·2 Z-scores for Pakistani infants (Figure 3). The accelerated growth in weight of Pakistani infants, relative to White infants, after 12 months, resulted in no significant differences in BMI between ethnic groups at 24 months of age (p-values > 0·4).

### Obesity risk

The ability of a growth chart to identify infants at increased risk of obesity is fundamentally important. In the context of a new growth chart, it is important to understand the differences that such a chart might make, compared to any previously used chart, because of the different source sample rather than because of any actual change in risk in the population. In comparison to the UK90 reference charts, the UK-WHO charts were significantly (p-values < 0.05) more likely to classify Bradford infants as overweight after six months of age (relative risks 1.427 to 2.151) and obese after eight months of age (relative risks 1·875 to 2·263) (Table 1). Further, the UK-WHO charts were significantly more likely to classify infants as demonstrating rapid or extremely rapid (i.e., > one or two centile bands, respectively) gains in weight and/or BMI (relative risks 1.214 to 1.470).

**Table 1:** **The risk of obesity risk traits**^**1**^**in 2181 infants**

	**UK-WHO charts**	**UK90 reference charts**	**Relative Risk (95% Confidence Interval)**
**Overweight (%)**			
1 month	2.2	2.5	0.889 (0.605, 1.305)
2 months	1.7	2.8	0.607* (0.405, 0.909)
3 months	2.4	3.2	0.768 (0.540, 1.093)
4 months	3.3	3.4	0.959 (0.697, 1.321)
5 months	3.9	3.5	1.132 (0.836, 1.532)
6 months	4.9	3.4	1.427* (1.068, 1.905)
7 months	6.7	4.0	1.670* (1.291, 2.161)
8 months	9.0	4.9	1.849* (1.471, 2.324)
9 months	11.5	6.0	1.931* (1.576, 2.366)
10 months	14.2	7.3	1.938* (1.616, 2.323)
11 months	17.2	9.0	1.909* (1.623, 2.244)
12 months	21.0	10.9	1.929* (1.668, 2.229)
24 months (n = 751)	24.6	11.5	2.151* (1.701, 2.721)
**Obesity (%)**			
1 month	0·3	0·4	0·750 (0·261, 2·158)
2 months	0·1	0·3	0·500 (0·125, 1·997)
3 months	0·2	0·2	1·000 (0·250, 3·993)
4 months	0·2	0·2	0·800 (0·215, 2·975)
5 months	0·5	0·3	1·429 (0·545, 3·746)
6 months	1·0	0·6	1·750 (0·863, 3·548)
7 months	1·2	0·8	1·500 (0·829, 2·715)
8 months	1·9	1·0	1·952* (1·158, 3·292)
9 months	2·8	1·5	1·875* (1·226, 2·868)
10 months	4·2	2·0	2·140* (1·497, 3·058)
11 months	6·0	2·7	2·241* (1·654, 3·037)
12 months	7·9	3·5	2·263* (1·739, 2·945)
24 months (n = 751)	9·2	4·4	2·091* (1·398, 3·126)
**Weight gain, 0–12 months**			
**Rapid (%)**	50.0	38.9	1.257* (1.181, 1.337)
**Extremely (%) rapid**	27.5	20.7	1.214* (1.124, 1.311)
**BMI gain, 1–12 months**			
**Rapid (%)**	55.2	36.5	1.470* (1.380, 1.567)
**Extremely rapid (%)**	32.6	19.8	1.440* (1.328, 1.561)

*significant at p < 0.05.

^1^Overweight, BMI > 1.34 Z-scores = 91^st^ centile; obesity, BMI > 2.05 Z-scores = 98^th^ centile; rapid weight or BMI gain, gain > 0.67 Z-scores = one centile band; extremely rapid weight or BMI gain, gain > 1.34 Z-scores = two centile bands.

The higher risk of overweight or obesity using the UK-WHO charts compared to the UK90 reference charts emerged one to two months earlier for White infants compared to Pakistani infants (Table 2); the risks of rapid and extremely rapid weight and BMI gain were similar for both ethnic groups.

**Table 2:** **The risk of obesity risk traits**^**1**^**in 1187 White and 994 Pakistani infants**

	**UK-WHO charts**		**UK90 reference charts**		**Relative Risk (95% Confidence Interval)**	
	**White**	**Pakistani**	**White**	**Pakistani**	**White**	**Pakistani**
**Overweight (%)**						
1 month	3.4	0.8	3.8	0.9	0.889 (0.585, 1.350)	0.889 (0.344, 2.295)
2 months	2.7	0.5	4.2	1.1	0.640* (0.414, 0.990)	0.455 (0.159, 1.303)
3 months	3.7	0.9	4.7	1.3	0.786 (0.534, 1.157)	0.692 (0.297, 1.612)
4 months	4.7	1.5	5.0	1.5	0.949 (0.664, 1.356)	1.000 (0.492, 2.035)
5 months	6.0	1.5	5.1	1.5	1.164 (0.835, 1.623)	1.000 (0.492, 2.035)
6 months	7.1	2.3	5.1	1.5	1.400* (1.015, 1.931)	1.533 (0.805, 2.921)
7 months	9.4	3.5	5.8	1.9	1.623* (1.216, 2.167)	1.842* (1.061, 3.198)
8 months	12.0	5.4	6.7	2.6	1.775* (1.366, 2.306)	2.077* (1.312, 3.288)
9 months	15.1	7.2	7.9	3.6	1.904* (1.503, 2.412)	2.000* (1.354, 2.955)
10 months	18.4	9.2	9.8	4.4	1.888* (1.530, 2.330)	2.068* (1.459, 2.932)
11 months	21.9	11.7	12.0	5.5	1.831* (1.517, 2.210)	2.109* (1.549, 2.872)
12 months	26.1	15.0	14.1	7.1	1.856* (1.566, 2.200)	2.099* (1.604, 2.745)
24 months (n = 332 White, 419 Pakistani)	24.7	24.6	11.1	11.7	2.216* (1.551, 3.167)	2.102* (1.539, 2.872)
**Obesity (%)**						
1 month	0·4	0·1	0·6	0·1	0·714 (0·227, 2·244)	1·000 (0·063, 15·965)
2 months	0·2	0·1	0·4	0·1	0·400 (0·078, 2·058)	1·000 (0·063, 15·965)
3 months	0·3	0·1	0·3	0·1	1·000 (0·202, 4·945)	1·000 (0·063, 15·965)
4 months	0·3	0·1	0·3	0·1	0·750 (0·168, 3·344)	1·000 (0·063, 15·965)
5 months	0·7	0·2	0·5	0·1	1·333 (0·464, 3·831)	2·000 (0·182, 22·021)
6 months	1·4	0·4	0·7	0·4	2·125 (0·921, 4·905)	1·000 (0·251, 3·987)
7 months	1·9	0·4	1·2	0·4	1·643 (0·850, 3·177)	1·000 (0·251, 3·987)
8 months	3·0	0·5	1·4	0·4	2·118* (1·196, 3·749)	1·250 (0·337, 4·641)
9 months	4·2	1·0	2·4	0·4	1·786* (1·132, 2·816)	2·500 (0·787, 7·945)
10 months	5·7	2·4	3·2	0·5	1·789* (1·213, 2·640)	4·800* (1·839, 12·530)
11 months	7·6	4·0	4·2	0·8	1·800* (1·286, 2·519)	5·000* (2·352, 10·627)
12 months	10·1	5·2	5·2	1·4	1·935* (1·440, 2·602)	3·714* (2·072, 6·657)
24 months (n = 332 White, 419 Pakistani)	8·4	9·8	4·2	4·5	2·000* (1·072, 3·730)	2·158* (1·274, 3·654)
**Weight gain 0–12 months**						
**Rapid (%)**	47.3	53.2	36.5	41.8	1.254* (1.151, 1.366)	1.262* (1.153, 1.381)
**Extremely (%) rapid**	24.6	30.9	18.1	23.8	1.228* (1.099, 1.371)	1.203* (1.081, 1.340)
**BMI gain 1–12 months**						
**Rapid (%)**	54.4	56.0	35.5	37.8	1.485* (1.361, 1.621)	1.454* (1.324, 1.595)
**Extremely rapid (%)**	31.9	33.4	19.5	20.0	1.427* (1.278, 1.593)	1.456* (1.292, 1.641)

*significant at p < 0.05.

^1^Overweight, BMI > 1.34 Z-scores = 91^st^ centile; obesity, BMI > 2.05 Z-scores = 98^th^ centile; rapid weight or BMI gain, gain > 0.67 Z-scores = one centile band; extremely rapid weight or BMI gain, gain > 1.34 Z-scores = two centile bands.

## Discussion

This paper investigated childhood obesity risk using the UK-WHO charts in a sample of normal UK infants not selected on the basis of any defining characteristics. Our primary finding was that UK infants, on average, demonstrated a striking pattern of accelerated BMI growth against the UK-WHO charts. That pattern was characterised by an average gain between one and 12 months of age of 1.0 BMI Z-score, equivalent to upward crossing through approximately 1.5 centile bands (e.g., 50^th^ centile to the 85^th^ centile). Subsequently, we observed a greater risk of high infant BMI values and upward centile crossing using the UK-WHO charts compared to the previously used UK90 growth reference charts. The study design, which compared one sample of infants born at one point in time (2008–2009) to two different growth charts, means that the presented differences in obesity risk were entirely due to difference between the charts (e.g., source sample, statistical design) not because of any change in risk within the population. Our findings highlight the fact that the source sample of a growth chart has a fundamentally important role to play in childhood obesity risk classification; the switch from the UK90 reference to the UK-WHO charts will result in more infants being diagnosed as being on a trajectory toward childhood obesity. Indeed, combined data from the 2008/2009 and 2009/2010 National Diet and Nutrition Survey (NDNS) in the UK showed an obesity prevalence in two to three year old infants almost double that found in children aged four to 10 years [25], assumedly because the infants were assessed using the UK-WHO charts whilst the children were assessed using the UK90 reference, not because of any real change in risk within the population. This is a perfect example of how the introduction of the UK-WHO charts may lead to misinterpretation of obesity risk in the UK.

Breastfed infants demonstrate slower growth than their bottle-fed counterparts [14,16], with evidence suggesting that this may be because they are better at self-regulating their total energy intake [26,27]. According to national statistics (13), the average infant in the UK does not follow the WHO feeding regime of exclusive breastfeeding to at least four months of age [10]. When their growth is assessed against a chart based on data from infants who did follow the WHO feeding regime, they therefore demonstrate a pattern of accelerated growth indicative of increased risk for childhood obesity. This rationale is supported by the available literature, which shows that the choice to bottle-feed rather than breastfeed contributes most to accelerated infant growth [28-30], which in turn contributes most to the development of childhood obesity [5].

The Z-scores of White infants in the present study roughly approximate to those observed by Wright et al. [17], who used data from the Avon Longitudinal Study of Parents and Children (ALSPAC) and the Gateshead Millennium baby Study (GMS) to test the WHO standard in the UK, at the cross-sectional ages where those researchers had data. The main strengths of the present study are that we modelled longitudinal data to supply continuous comparative information and assessed the risk of childhood obesity risk traits not included in the Wright et al. paper. The fact that we compared to the UK-WHO charts and not the WHO standard is actually of little importance because the only difference would have occurred at birth, where the UK-WHO charts are based on recalculated UK90 reference data [12]. If anything, using the WHO standard data at birth instead of recalculated UK90 reference data would have resulted in a lower degree of subsequent accelerated weight gain because, in the present study, birth weight Z-scores according to the WHO standard were approximately 0.2 units higher than those using the recalculated UK90 reference data (mean values for White infants (sexes combined) +0.22 and −0.04, respectively). Wright et al. [17] found that ALSPAC and GMS infants appeared large at birth using the WHO standard and focused on that finding, but not on the subsequent apparent accelerated growth which has major implications on the interpretation of childhood obesity risk in the UK.

The present paper helps inform the practitioner, who ultimately faces the interpretive dilemma of wondering whether a specific growth pattern should give cause for concern, about average growth patterns and risks of childhood obesity according to the UK-WHO charts. The correct interpretation of apparent accelerated growth, however, presupposes that the practitioner knows that the UK-WHO chart is essentially a growth standard, knows the differences between a standard and a reference, and knows the difference in how to interpret growth when using a standard compared to when using a reference. Because the number of UK infants who are diagnosed as being on a trajectory toward childhood obesity must have increased since the introduction of the UK-WHO charts, an increased effort to integrate the dissemination of useful information to parents with growth monitoring practice is necessary.

Not being able to test the relative contribution of factors responsible for accelerated growth of UK infants against the UK-WHO charts is perhaps the greatest limitation of the present study, because it would have allowed us to definitively answer the question “why do UK infants demonstrate obesogenic growth trajectories (against the UK-WHO charts)”?. Other limitations include a sample composed exclusively of infants from one city in the UK, which may limit generalisability of the results, and weight and BMI gain variables that did not account for regression to the mean. Gain variables would normally be calculated as the residuals from the general linear regression of size at age “T” on size at age “T-1” [24]. However, the relative risk analysis in the present study needed to include data from the same individuals against the UK-WHO charts and against the UK90 reference charts, so any initial regression would need to account for this non-independence; no formula has yet been proposed.

## Conclusions

Because normal UK infants demonstrate accelerated weight but not length growth against the new growth charts in the UK, the UK-WHO charts, they risk being diagnosed as being on a trajectory toward childhood obesity. According to the present study, twice as many UK infants may meet the criteria used to define overweight and obesity today compared to three years ago when the UK90 reference was the chart of choice. National estimates of overweight and obesity will have to be recalculated for previous years to allow longitudinal comparison. The new charts do “support efforts to avoid future childhood obesity” [17], but this is irrelevant if practitioners have not received adequate training on how to interpret growth against a prescriptive standard and if support mechanisms for parents with an infant diagnosed as “high risk for childhood obesity” are not in place. The UK-WHO charts necessarily do not allow a focused prevention effort for targeting programmes at infants most at risk of becoming obese, because the use of the 91^st^ or 98^th^ centile on the UK-WHO charts will identify many more infants as being at risk than the same centiles on the UK90 reference. Now more than ever, research is needed to develop a large scale childhood obesity prevention programme which could be integrated with routine infant growth monitoring practice.

## Abbreviations

UK, United Kingdom; WHO, World Health Organisation; MNI, Body mass index; ALSPAC, Avon longitudinal study of parents and children; GMS, Gateshead millennium baby study; NDNS, National diet and nutrition survey.

## Competing interests

The authors declare that they have no competing interests.

## Authors’ contributions

WJ designed research, performed statistical analysis, and wrote paper. NC was responsible for project conception, development of overall research plan, and had primary responsibility for final content. JW interpreted data and revised manuscript. All authors provided critical revision for important intellectual content and approved the final version.

## Pre-publication history

The pre-publication history for this paper can be accessed here:

http://www.biomedcentral.com/1471-2431/12/104/prepub
